# Two cases of perivenous hepatic iron deposition in the background of alcoholic liver cirrhosis

**DOI:** 10.1007/s00261-024-04624-x

**Published:** 2024-10-12

**Authors:** Takahiro Komori, Norihide Yoneda, Kazuto Kozaka, Naoki Ohno, Noboru Takata, Akihiro Seki, Kaori Yoshimura, Hiroko Ikeda, Kenichi Harada, Satoshi Kobayashi

**Affiliations:** 1 Department of Radiology, Kanazawa University Graduate School of Medical Sciences, Kanazawa, Japan; 2 Faculty of Health Sciences, Institute of Medical, Pharmaceutical and Health Sciences, Kanazawa University, Kanazawa, Japan; 3 Department of Gastroenterology, Kanazawa University Graduate School of Medical Sciences, Kanazawa, Japan; 4 Department of Human Pathology, Kanazawa University Graduate School of Medical Sciences, Kanazawa, Japan; 5 Department of Pathology Kanazawa University Hospital, Kanazawa, Japan

## Introduction

Hepatic iron deposition in liver cirrhosis is typically seen as uniform iron deposition or iron-containing nodules on MRI. While liver biopsy has traditionally been used for diagnosis, advances in MRI, such as R2* mapping images with iterative decomposition of water and fat with echo asymmetry and least squares estimation quantification sequence (IDEAL-IQ) or modified Dixon chemical-shift imaging Quant (mDIXON Quant), allow non-invasive assessment of liver iron [1, 2]. Imaging-based detection of iron deposition helps diagnose the underlying causes of iron overload. Quantitative assessment methods for hepatic iron deposition have also been reported [3, 4]. In rare cases, iron preferentially deposits around hepatic veins in liver cirrhosis with iron overload. Fat deposition around hepatic veins is known in alcoholic liver disease [5], and similar mechanisms causing iron deposition around hepatic veins have also been reported, though infrequently [6–8]. We report two cases of perivenous hepatic iron deposition in patients with alcoholic cirrhosis, hemolytic anemia and iron overload, focusing on MRI and pathological findings.

## Case reports

### Case 1

**Fig. 1:**
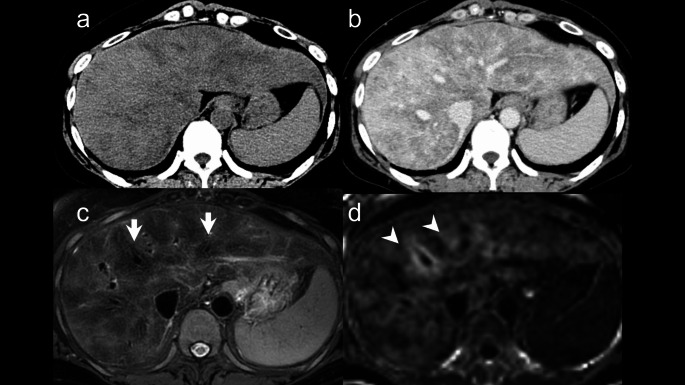
Image and histological findings of case 1. (**a**) Plain CT: Diffuse ill-defined low-density areas were observed. (**b**) Equilibrium Phase with Contrast: Heterogeneous low-attenuation areas were noted around the Glisson’s sheath and hepatic veins.　(**c**) FST2WI: Prominent low-signal areas were observed around the hepatic veins (arrows).　(**d**) R2*map: High values were detected around the hepatic veins (arrowheads), suggesting iron deposition in the hepatic parenchyma

A 54-year-old female, who was diagnosed with alcoholic liver disease 8 years ago due to liver dysfunction and abdominal distension was advised to quit alcohol consumption. Two years prior, she experienced worsening joint pain and visited a local clinic. During this visit, liver dysfunction, decreased platelet count, and complement deficiency were observed, prompting a referral to our hospital for a comprehensive evaluation. Upon evaluation at our hospital, the patient exhibited liver dysfunction, decreased platelet count, and elevated indirect bilirubin levels. Further diagnostic workup revealed evidence of hemolysis, including low haptoglobin and elevated reticulocyte count, confirming a diagnosis of hemolytic anemia in addition to hypersplenism. During her hospitalization, her liver function showed improvement. Her drinking history was approximately 14 standard drinks per day in her late thirties. The laboratory data are summarized in Table 1. Detailed examinations, including contrast-enhanced dynamic computed tomography (CT) and gadolinium-ethoxybenzyl-diethylenetriamine-pentaacetic acid-enhanced MRI (Gd-EOB-DTPA-enhanced MRI) were performed to evaluate liver function disorders. Plain CT revealed a diffuse, ill-defined hypodense area within the liver parenchyma, and contrast-enhanced CT revealed non-uniform hypodense areas around the Glisson’s sheath and hepatic veins (Fig. 1a, b). On MRI, the liver parenchyma exhibited non-uniform signal intensity on fat-suppression T2-weighted image (FST2WI), with a prominent low-signal area around the hepatic veins (Fig. 1c). In the hepatobiliary phase (HBP), the liver parenchyma showed a low signal intensity in the same region and R2* mapping indicated elevated values, suggesting iron deposition (Fig. 1d). Fat Fraction mapping revealed a mildly high signal area around the hepatic veins, indicating mild fat deposition. Transjugular liver biopsy (TJLB) was performed to investigate the cause of the hepatic disorder. Histologically, the lobular architecture of the liver was disrupted, and significant fibrosis was observed around the portal areas in a perilobular pattern and around the hepatocytes, often associated with bridging fibrosis. Approximately 10% of the liver parenchyma exhibited fat deposition and features such as ballooning hepatocytes, nuclear glycogen, and focal necrosis were scattered throughout. This condition was considered consistent with alcoholic steatohepatitis and pre-cirrhotic liver changes. Iron staining revealed moderate deposition of positive granules within hepatocytes.

**Table 1:** Laboratory data

Table 1	Case 1	Case 2	Normal range
Hb (g/dL)	8.0	6.6	11.6–14.8
Plt	61 × 10^3^	64 × 10^3^	158–348
PT (seconds)	20.5	22.8	10.5–13.0
PT Activity (%)	37	32	78.7–123.0
APTT (seconds)	46.6	50.0	24.0–34.0
ALP (IU/L)	114	200	38–113
γ-GTP (IU/L)	68	25	10–47
AST (IU/L)	118	77	13–33
ALT (IU/L)	53	52	6–27
T-bil (mg/dL)	10.6	15.4	0.3–1.2
D-bil (mg/dL)	3.9	5.0	< 0.5
Alb (g/dL)	3.2	2.5	4.0–5.0
AFP (ng/mL)	136	36	< 10
PIVKA II (mAU/mL)	83	269	< 40
Ferritin (ng/mL)	931	1855	6.23–138

Hb, hemoglobin; Plt, platelets; PT, Prothrombin time; APTT, activated partial thromboplastin time; ALP, alkaline phosphatase; γGTP, gamma-glutamyl transpeptidase; AST, aspartate transaminase; ALT, alanine transaminase; T-bil, total bilirubin; D-bil, direct bilirubin; Alb, albumin; AFP, alpha fetoprotein; PIVKA-2, protein induced by vitamin K absence/antagonist-II

### Case 2

**Fig. 2:**
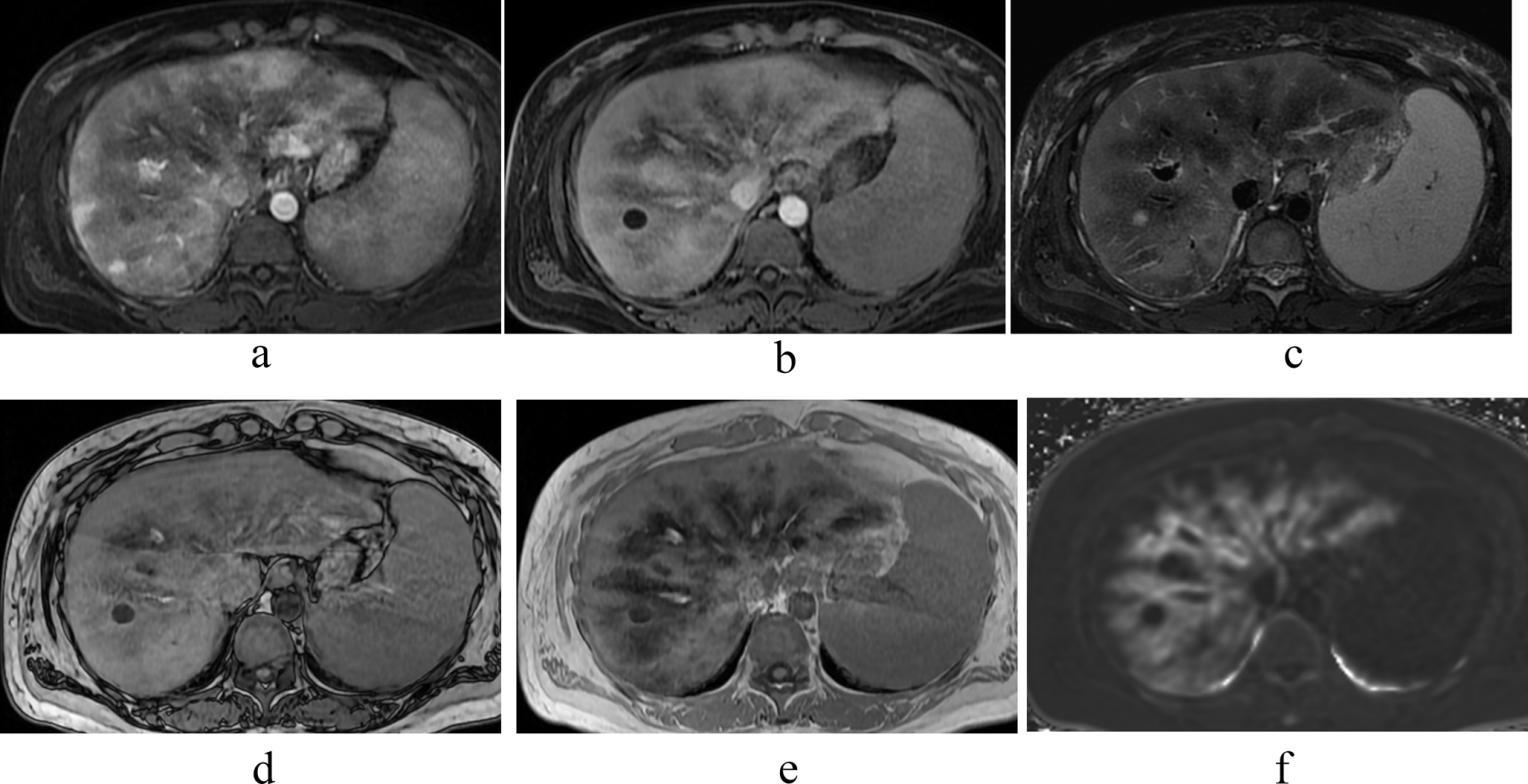
Image findings of case 2. (**a**) Contrast T1WI (arterial phase): Heterogeneous enhancement was observed.　(**b**) EOB HBP: Prominent low-signal areas were observed around the hepatic vein. (**c**) FST2WI: The same area exhibited a low signal, suggesting iron deposition. (**d**) T1WI Out of Phase and (**e**) T1WI In Phase: The liver parenchyma around the hepatic veins showed signal reduction extending from out of phase to in phase T1WI because of TE prolongation. (**f**) R2*map: High values were detected around the hepatic vein

**Fig. 3:**
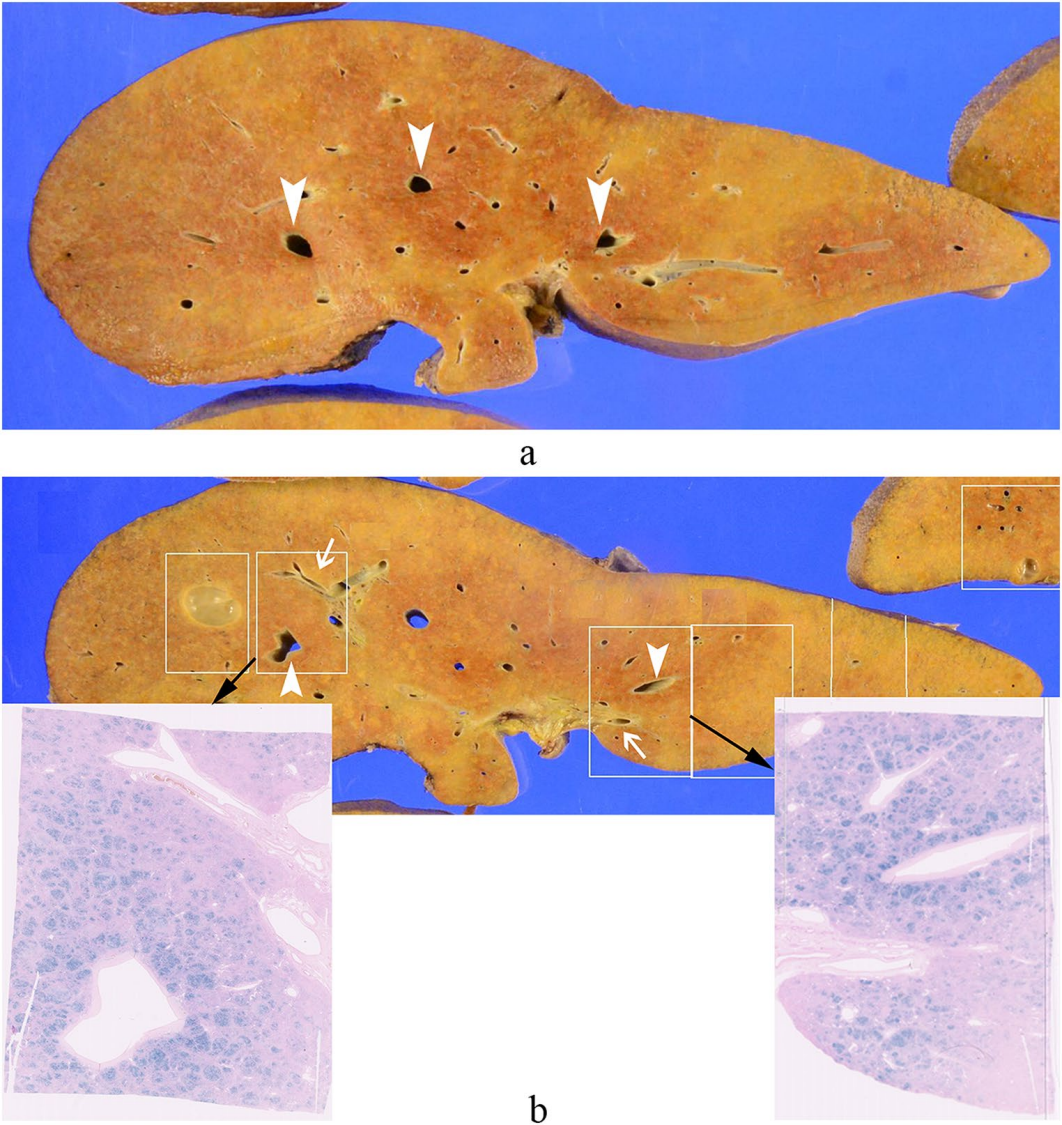
Pathological findings of case 2. (**a**) Macroscopically, the liver surface exhibited mild irregularities, and the cut surfaces displayed a range of colors from yellow to reddish-brown tones. Prominent reddish-brown areas were observed around the hepatic veins (indicated by arrowheads).　(**b**) Comparing the gross findings with the iron staining images, the reddish-brown areas that raised suspicion of iron deposition were more prominent around the large hepatic veins (arrowheads) compared to the areas around the portal vein (arrows)

A 45-year-old female with daily alcohol consumption was diagnosed 8 years ago with depression, alcoholism, and alcoholic cirrhosis after presenting with hallucinations. Over time, her liver function worsened. One year ago, she developed progressive anemia, elevated indirect bilirubin, and low haptoglobin, along with an increase in reticulocytes. After further evaluation by the hematology department, she was diagnosed with spur cell hemolytic anemia associated with alcoholic cirrhosis, and transfusion therapy was initiated. As her liver function worsened, she was referred to our hospital for a brain-dead liver transplant. Pre-transplant contrast-enhanced CT and MRI were conducted. A suitable donor was found during hospitalization, and the transplant was performed. The laboratory data are in Table 1. She had a drinking history of about seven standard drinks per day. Plain CT revealed heterogeneous hypodense areas in the hepatic parenchyma. Contrast-enhanced dynamic CT in the arterial phase revealed a heterogeneous pattern of hypodense areas within the liver parenchyma, whereas in the equilibrium phase, the liver parenchyma showed uniform enhancement. On MRI, the dynamic study findings were showing nonuniform enhancement within the liver parenchyma and prominent areas of poor enhancement around the veins. In the HBP, the same area showed a low signal intensity. The liver parenchyma around the hepatic veins showed signal reduction extending from out-of-phase to in-phase T1-weighted images (T1WI) because of echo time (TE) prolongation, and R2* mapping indicated elevated values, suggesting iron deposition. The same area exhibited low signal intensity on FST2WI (Fig. 2a-f). Pathological findings of the excised liver for brain-dead liver transplantation showed that, grossly, the liver surface had mild irregularities, with a cut surface displaying a yellow to reddish-brown hue and reddish-brown areas prominent around the hepatic veins. Histologically, small regenerative nodules surrounded by fibrous septa were diffusely observed, showing the image of liver cirrhosis. Prominent regenerative nodules with hemosiderin deposition were also observed. The findings were consistent with cirrhosis caused by alcoholic liver disease. Iron staining revealed that the reddish-brown pigment stained blue, indicating a state of hemosiderosis. The reddish-brown areas suspected of iron deposition were grossly prominent around the large hepatic veins, and a significant iron deposition pattern was observed in the same region (Fig. 3).

## Discussion

Diagnosing hepatic iron deposition usually requires a liver biopsy to evaluate excess iron. However, advances in MRI have made non-invasive assessment possible [1–4]. Typically, hepatic iron deposition on MRI appears as diffuse and homogeneous, but rare cases of “Perivenous hepatic iron deposition,” where iron accumulates around hepatic veins, have been reported [6–8]. Few studies have addressed heterogeneous iron deposition in the liver. Kawamori et al. reported a case of segmental iron deposition near a tumor-thrombosed intrahepatic portal vein, suggesting that reduced portal blood supply caused the wedge-shaped spread of iron deposits distal to the tumor thrombus [9]. Kadoya et al. reported cases of regional iron deposition in areas with reduced portal venous perfusion caused by portal vein thrombosis, tumor thrombus, arterioportal shunts, and tumor compression of the portal vein branch [10]. These findings suggest that reduced portal flow may lead to heterogeneous iron deposition.

In our two cases, both patients had alcoholic cirrhosis, hemolytic anemia, and elevated serum ferritin, indicating possible iron overload. Imaging showed splenomegaly, mild collateral circulation, and ascites, suggesting portal hypertension. MRI revealed dominant iron deposition around the hepatic veins, similar to previous reports [6–8]. Furthermore, in one case of liver extracted during brain-dead liver transplantation, reddish-brown areas were observed around the large hepatic veins. Iron staining revealed significant iron deposition in these areas. These findings suggest that a combination of alcoholic cirrhosis, reduced portal blood flow, and iron overload may lead to predominant iron deposition around the hepatic veins. Generally, hepatic iron overload in hereditary hemochromatosis or secondary hemosiderosis is characterized by increased liver attenuation on CT due to diffuse iron deposition. However, in our cases, no significant high attenuation was observed around the hepatic veins. It is known that in alcoholic cirrhosis, fat deposition around the hepatic veins can occur [5, 11], which was considered as a potential factor. Nevertheless, while fat deposition around the hepatic veins was noted in Case 1, it was less evident in Case 2. Therefore, this might be due to the milder iron deposition in alcoholic cirrhosis compared to typical iron overload disorders such as hereditary hemochromatosis and secondary hemosiderosis, but this should warranting further investigation.

In conclusion, patients with alcoholic cirrhosis, reduced portal blood flow, and iron overload may show characteristic iron deposition around the hepatic veins on MRI. Recognizing these findings can assist in non-invasive patient assessment.

## Data Availability

No datasets were generated or analysed during the current study.

## Abbreviations

MRI: Magnetic resonance imaging
T2WI: T2-weighted image
T1WI: T1-weighted image
TE: Echo time
IDEAL-IQ: Iterative decomposition of water and fat with echo asymmetry and least squares estimation quantification sequence
mDIXON Quant: Modified Dixon chemical-shift imaging Quant
CT: Computed tomography
Gd-EOB-DTPA-enhanced MRI: Gadolinium-ethoxybenzyl-diethylenetriamine-pentaacetic acid enhanced MRI
FS: Fat suppression
HBP: Hepatobiliary phase
TJLB: Transjuglar liver biopsy
HE: Hematoxylin-Eosin
